# Long-term psychological effects of war trauma and migration: an interpretative phenomenological analysis of Balkan war survivors

**DOI:** 10.1186/s40359-026-04033-3

**Published:** 2026-01-29

**Authors:** Markéta Nečasová, Marek Preiss, David Ulčák, Ivan Rektor, Alice Prokopová, Monika Fňašková

**Affiliations:** 1 Central European Institute of Technology (CEITEC), Centre for Neuroscience, Masaryk University, Brno, Czech Republic; 2 National Institute of Mental Health, Klecany, Czech Republic; 3 First Department of Neurology, Faculty of Medicine, St Anne’s University Hospital, Masaryk University, Brno, Czech Republic; 4 University of New York in Prague, Prague, Czech Republic; 5 Department of Health Education, Faculty of Education, Masaryk University, Brno, Czech Republic

**Keywords:** Balkan war survivors, War-related trauma, Emigration, Interpretative phenomenological analysis

## Abstract

**Background:**

The wars following the breakup of Yugoslavia led to mass displacement, violence, and long-term psychological suffering among civilians. While clinical responses to war-related trauma and refugee experiences have been widely studied, less is known about how survivors make sense of these events decades later and how trauma and emigration continue to shape their identity, relationships, and wellbeing.

**Objective:**

This study examines the long-term psychological impacts of war and migration among civilian survivors of the Balkan wars resettled in the Czech Republic, with attention to meaning-making processes decades after the original events.

**Method:**

We conducted an interpretative phenomenological analysis of in-depth, semi-structured interviews with four adult civilian survivors of the Balkan wars, all of whom experienced emigration and long-term resettlement.

**Results:**

Participants described wartime life as isolating, marked by survival-focused coping. They highlighted the importance of close relationships, routines, and developmental stage in shaping how they endured this period. Decades later, they reported persistent vigilance, moral reflection, and existential questioning, alongside posttraumatic growth. Migration was perceived as a prolonged, transformative process reshaping identities and relationships to cultural roots. Intergenerational impact emerged, with participants reflecting on survival strategies transmitted to their children.

**Conclusion:**

The findings suggest that war trauma and migration are not discrete events but temporally extended, relational processes, unfolding across the lifespan. This perspective advances psychological understanding of trauma trajectories, identity reconstruction, and intergenerational adaptation.

**Supplementary Information:**

The online version contains supplementary material available at 10.1186/s40359-026-04033-3.

## Background

Beginning in 1991, a series of armed conflicts spread across the former Yugoslavia, affecting Slovenia, Croatia, Bosnia and Herzegovina, and later Kosovo, culminating in the NATO bombing of Serbia and Montenegro in 1999. The Balkan wars led to large-scale violence, displacement, and persecution, profoundly affecting civilian populations. In Bosnia and Herzegovina, a region particularly affected by the violence, tens of thousands lost their lives as a result of armed hostilities and systematic acts of ethnic cleansing [1]. Furthermore, approximately two million people from Yugoslavia were internally displaced, while an estimated 1.2 million fled the region and sought refuge abroad [1, 2].

Civilians were frequently exposed to a wide range of potentially traumatic events, often experiencing multiple incidents over the course of the war [3–5]. The most commonly reported experiences were exposure to shelling and bombing, lack of shelter, and forced displacement from one’s home [6].

Such experiences have been consistently associated with elevated prevalence rates of posttraumatic stress disorder (PTSD), depression, anxiety disorders, somatisation, and paranoid ideation [4, 7]. Notably, these psychological symptoms are not limited to the immediate aftermath of conflict; rather, they may fluctuate in intensity over time [3] and, as evidenced in studies of Holocaust survivors, can persist for several decades [8, 9].

The impact of war extends beyond clinical symptoms, affecting cognitive and emotional processing, interpersonal interactions and sensitivity, overall life satisfaction, and mental health – ultimately shaping the everyday lives of survivors and their offspring [3, 4, 10–12].

The forced displacement of hundreds of thousands from the former Yugoslavia further compounded these psychological burdens, separating individuals from their homes, families, communities, and cultural identities [13]. This loss of place and identity often required a re-evaluation of fundamental beliefs about life, social roles, and self. All phases of the migration process – including pre-migration trauma, flight, and resettlement – were associated with heightened risk of both traumatisation and re-traumatisation [14].

In addition to psychological and psychiatric conditions, refugees faced elevated risks of chronic physical illnesses, such as cardiovascular diseases and cancer, underscoring the broad health impact of war trauma. Furthermore, post-migration stressors, including legal insecurity, limited economic opportunities, and perceived social exclusion, have been identified as compounding factors that may exacerbate psychopathological symptoms [10, 15]. Reported psychosocial difficulties include strained interpersonal relationships, feelings of inner insecurity and uprootedness, and distrust in institutions [16]. The psychological impact of war thus extends beyond active conflict, and it is often prolonged and intensified by adverse post-migration conditions. Individuals who returned to the Balkans appeared particularly vulnerable, with studies showing increased distress over time [3].

While quantitative research has substantially advanced understanding of prevalence rates and risk factors, less is known about how survivors interpret and integrate war-related trauma into their lives over time. Most studies focus on diagnosis and epidemiology, often overlooking the subjective, evolving processes of meaning-making. Yet, meaning-making is central to understanding how individuals reconstruct identity and continuity after adversity.

The concept of posttraumatic growth provides one lens for examining such processes. Rather than viewing growth as a linear or fixed outcome, contemporary models frame it as a dynamic process shaped by personal history, social context, and developmental stage [17, 18]. Growth and distress often coexist within the same narrative, as individuals attempt to reconstruct meaning and life trajectories.

Despite extensive research on psychopathology, little is known about how war-related trauma interacts with long-term migration processes, such as loss of homeland, identity reconstruction, and adaptation to new sociocultural contexts. This study addresses this gap by examining the lived experiences of individuals who survived the Balkan wars and resettled abroad. Using Interpretative Phenomenological Analysis (IPA) and semi-structured interviews, we explore how participants make sense of war and migration as an interconnected process that continues to shape their everyday lives, identity, and sense of security.

## Methods

### Study design and setting

This qualitative study employed Interpretative Phenomenological Analysis (IPA) to explore lived experiences. The research was conducted between 2022 and 2025 at the Central European Institute of Technology – Research Centre of Masaryk University in Brno. It was approved by the Ethics Committee of Masaryk University in accordance with the Declaration of Helsinki. The ethics committee’s approval code number is EKV-2021-076. Informed consent was obtained from each participant. Participants were informed about confidentiality procedures, their right to withdraw at any time, and were offered the opportunity to consult with a psychotherapist.

### Data collection

Participants were recruited between 2022 and 2024 through multiple channels, including media coverage, university information platforms, social networks, and a public lecture at the Lastavica club, a cultural organisation that brings together members of the Balkan diaspora in the Czech Republic. For the in-depth interview phase, only individuals who had explicitly consented with the follow-up and who met the inclusion criteria (having experienced the Balkan wars during adolescence or young adulthood and eventually resettled in the Czech Republic) were invited to take part in the in-depth interview phase conducted between January and March 2024. A purposive sampling strategy was therefore applied to ensure relevance and depth of lived experience.

Data were collected through semi-structured, in-depth interviews exploring participants’ wartime and migration experiences and the long-term psychological and relational impacts. The interview guide, developed specifically for this study, covered (a) wartime experiences and coping, (b) migration and resettlement trajectories, (c) identity and relationships, and (d) perceived long-term effects. The guide was developed by the first author and refined through discussion with two co-authors. The final version of the interview guide is available as supplementary material (*Additional file 1 – Interview guide*). Before each interview, participants were informed about the study’s aims and the interviewer introduced herself and her interest in the topic. The interviewer had no personal experience with the Balkan wars.

Interviews lasted 35 to 105 min and were conducted in a one-on-one setting, either face-to-face or via video call, depending on participant preference. Two interviews were conducted in Czech and two in English. All interviews were audio-recorded with consent and transcribed verbatim. Selected Czech excerpts were translated into English for publication purposes.

### Participants

To ensure a relatively homogeneous sample consistent with IPA guidelines, the following inclusion criteria were applied: (a) civilian experience of the 1990s armed conflicts in former Yugoslavia, (b) emigration to the Czech Republic (directly or via other countries), and (c) being an adolescent or a young adult during the war.

The final sample comprised four participants: three females and one male (see Table 1 for demographic details). Although the sample size is at the lower end of what is commonly reported in IPA studies, it is well aligned with IPA’s idiographic commitment to in-depth exploration of lived experience within a relatively homogeneous group [19, 20]. The focus on depth rather than breadth enabled a detailed, case-by-case analysis while maintaining analytic feasibility [20]. The deliberate homogeneity in developmental stage and subsequent resettlement in the same host country further increased the information power of the sample for the specific research question [21]. In keeping with established IPA practice, theme sufficiency was reached by the fourth interview: no new superordinate themes emerged after the third participant, and the fourth interview primarily served to refine and deepen the existing five superordinate themes.

At the time of the interviews, participants were between 43 and 55 years of age (M = 47.5, SD = 4.56). Three participants lived in the former Yugoslavia during the war; one emigrated at its onset. Among those who remained, one experienced the war in Bosnia and Herzegovina, as well as the 1999 NATO bombing; two experienced the war’s economic aftermath and the 1999 bombing in Serbia.

**Table 1:** Characteristics of the participants (names have been anonymised)

	Simone	Gabrielle	Radek	Linda
Sex	Woman	Woman	Man	Woman
Age	55	45	47	43
Highest education	PhD	Master’s degree	High school with diploma	PhD
Marital status	Divorced	With partner	Single	Married
Interview language	Czech	English	English	Czech
Interview form	Video call	Video call	Face to face	Face to face

### Data analysis

Interview transcripts were analysed using Interpretative Phenomenological Analysis (IPA), an inductive, idiographic method grounded in phenomenology and hermeneutics [19, 20]. IPA embraces a double hermeneutic: participants make sense of their experiences and researchers interpret that meaning-making. This dual process enables in-depth exploration of meaning-making, balancing both an empathic understanding and a critical analysis of the respondent’s account.

The lead researcher engaged in reflexive practice prior to and during analysis, to identify assumptions, emotional responses, and potential interpretative biases. The analysis followed the recommended six-step IPA process: (1) repeated reading and exploratory commenting at descriptive, linguistic, and conceptual levels; (2) development of emergent themes; (3) clustering into subordinate themes; (4) production of a structured table of superordinate themes for each individual case; (5) cross-case analysis to identify convergent and divergent patterns; and (6) final integration into the five group-level superordinate themes presented in Table 2. The first author led the analysis, which was reviewed and discussed with two co-authors to support critical scrutiny of emerging interpretations and to enhance reflexivity and rigour. A detailed illustrative example of the analytic progression from raw transcript data to exploratory comments, emergent themes, subordinate themes, and final superordinate theme is provided as supplementary material *(Additional file 2 – Illustrative example of the IPA analytic process)*.

## Results

The analysis revealed five superordinate experiential themes. These are: *(1) We survived that*,* but we did not thrive*,* (2) Protective factors during wartime*,* (3) Enduring imprints of war*,* (4) Relating to one’s roots*,* (5) Transgenerational transmission.* See Table 2 for a summary of superordinate themes and subordinate themes.

**Table 2:** Summary of superordinate and subordinate experiential themes

Superordinate themes and subordinate themes		*n* of participants contributing and their names
We survived that, but we did not thrive		
	Loss of security and personal safety	4 (All)
	Adaptation as a way of life	4 (All)
	Isolation and estrangement	4 (All)
	Longing for it to end	3 (Gabrielle, Linda, Simone)
Protective factors during wartime		
	Not being on your own	4 (All)
	Maintaining a sense of normalcy	2 (Gabrielle, Radek)
	It was worse for parents	3 (Gabrielle, Linda, Radek)
Enduring imprints of war		
	Posttraumatic growth	4 (All)
	Desire for freedom	4 (All)
	Preparedness for emergencies	4 (All)
	Condemnation of war	4 (All)
	Haunting of the past	4 (All)
Relating to one’s roots		
	Ambivalent identity and the elusiveness of belonging	3 (Gabrielle, Radek, Simone)
	Emigration as opportunity and ambiguity	4 (All)
	The Balkans as an emotional and cultural anchor	2 (Linda, Simone)
Transgenerational transmission		
	Preparing for socio-political awareness	2 (Linda, Simone)
	Teaching practical survival skills	2 (Linda, Simone)

### Superordinate theme 1: we survived that, but we did not thrive

All participants described a profound disruption to their pre-war lives, consistently expressing they had ‘lost the life they had known’. Wartime was not perceived as a time of meaningful engagement with life, but as a phase dominated by basic survival, shaping participants’ subsequent life trajectories and decision-making in the post-war context.

#### Subordinate theme 1.1: loss of security and personal safety

A pervasive narrative was the experience of existential threat – a fundamental rupture of safety extending beyond physical danger. This disruption was experienced universally, whether individuals remained in the Balkans during the war or fled due to escalating violence. For Gabrielle, Radek and Linda, the perception of danger was rooted in the presence of a direct physical threat.

... *danger is coming from above; you don’t know when it’s going to come. You see people around you* *die*; *all of a sudden*,* they’re gone. And like… nothing can be done. (Radek)*

Although Simone experienced the conflict as a refugee, her narrative conveys a persistent sense of physical threat and existential disorientation, despite being physically removed from the war zone. Unlike others, her distress was further compounded by the loss of social securities, such as social capital, roles and status, commonly disrupted in forced migration.

*It was that feeling of completely like… nothing makes sense and they’re going to kill everybody in the end somehow… Even that feeling of physical threat was very intense*,* regardless of the fact that I was here and not there.*

#### Subordinate theme 1.2: adaptation as a way of life

Survival required resourceful strategies to navigate an unpredictable reality. Adaptation became a sustained mode of living, often forcing participants to set aside former interests, aspirations, or parts of their identity to focus on immediate survival.

It manifested in diverse ways: modifying recipes due to scarce ingredients, finding alternative forms of entertainment, or shifting career paths to meet basic survival needs.

... *I actually started a business*,* which was absolutely not something I was interested in*,* what I wanted to do*,* but it was necessary at the time to survive. (Simone)*

These adjustments were not seen as extraordinary resilience but as normalised routines. Gabrielle captured this paradox – what might seem extreme in hindsight had become ordinary at the time, leaving little space for reflection or resistance:

… *interesting about this experience is maybe how quickly we adapted and we found workarounds. Because right now when I tell you that I used to live for three months without electricity*,* we adapted and we functioned and it was ‘normal’.*

#### Subordinate theme 1.3: isolation and estrangement

Isolation emerged as another dominant experience, though its sources varied. Gabrielle reflected on how ethnic identity reshaped her relationship with once-familiar places:

… *even fairly close places were so far away. We lived very close to the Croatian border*,* and all of a sudden*,* this place became more far away than Australia to me – because I’m a Serb. I am an ethnic Serb.*

Simone’s isolation arose from being a newcomer in a foreign country, lacking social support and facing unprepared institutions. Linda and Radek, who stayed in Serbia, described political and cultural isolation due to sanctions and conflict. This sense of estrangement was compounded by material deprivation. The war imposed economic scarcity, limiting access to food, clothes and basic needs – a condition that later influenced participants’ values and behaviours in adulthood.

... *the first time I came [to the Czech Republic]*,* I had some of my own money*,* here I had the opportunity to buy more things*,* clothes… to have some meals*,* to choose what I wanted and not to think whether I had money for it. (Linda)*

#### Subordinate theme 1.4: a longing for it to end

When reflecting on what might have helped during the war, participants voiced one predominant wish: for the war to end. Survival had overtaken all other concerns and meaningful life was deferred until peace returned.

... *in order to really feel like being here*,* it was necessary for the war to end. Because it was just a permanent reason for terrible misfortune. (Simone)*

This statement captures the emotional weight of the wartime experience, reflecting a longing for normality defined primarily by the absence of fear and loss.

### Superordinate theme 2: protective factors during wartime

Amidst the pervasive uncertainty and threat of war, participants emphasised the stabilising role of social connections, daily routines, and developmental factors that helped them endure the psychological strain of war. Coping was not framed as a formal strategy but as a shared, improvised effort to maintain a sense of normalcy and belonging.

#### Subordinate theme 2.1: not being on your own

A shared element was the importance of facing the experience together rather than in isolation. In the absence of professional psychological services or systemic support, family and close friends became crucial sources of comfort.

... *we had to be self-sufficient with each other. You didn’t have a psychologist*,* a paediatrician*,* you know*,* whatever… we just all talked an awful lot about everything… (Linda)*.

#### Subordinate theme 2.2: maintaining a sense of normalcy

Ordinary routines, such as attending school sporadically or engaging in small daily activities, provided a semblance of order and distraction from danger:

… *you know*,* we go to school for a month*,* then we don’t go for*,* I don’t know*,* a month*,* then we go back. But I think this also kept the structure and this impression of normalcy. (Gabrielle)*

#### Subordinate theme 2.3: it was worse for parents

Participants also reflected on how age influenced the experience of war. Participants’ developmental stages shaped how they comprehended and coped with the events. Several expressed the belief that children or adolescents may have been shielded from the full existential weight of the situation, unlike adults who bore the responsibility for others.

*I think it’s somehow better for us younger ones; when you’re young*,* you take it differently than those parents who worry about the kids. (Linda)*

### Superordinate theme 3: enduring imprints of war

The war experience became internalised, continuing to shape participants’ identities, values, and worldviews in diffuse but lasting ways. Its impact was inseparable from their sense of self, often blurring where the war ended and personal identity began:

… *we will never know what I would do or who I would be if I didn’t have that experience*,* but now that I do have it*,* it* *did* *colour my life in a very specific colour. (Gabrielle)*

… *that experience changed me completely… the way I experience the world*,* the way I evaluate the events that happen in the world and in our country*,* you know*,* like a permanent threat that it can happen again. (Simone)*

The imprints were described as both constraining and transformative: a source of scars but also of growth, shaping values, priorities, and perceptions of threat. Participants described five domains in which these enduring imprints were most evident.

#### Subordinate theme 3.1: posttraumatic growth

Despite psychological scars, participants reported greater empathy, greater appreciation for close relationships, and stronger sense of resilience. Radek reflected on how his wartime experiences allowed him to comfort Ukrainian co-workers at the outbreak of their conflict:

… *it meant something that I’ve been through this experience… at least*. *I was hoping [I was] providing some kind of comfort to those kids at those moments as much as I could… (Radek)*.

#### Subordinate theme 3.2: desire for freedom

Experiences of war isolation and restrictions deepened participants’ appreciation of freedom and influenced their decision to emigrate. Gabrielle metaphorically described this impulse:

*I think that teenager actually said: OK*,* let’s go. Let’s go see. Let’s go now. Now you can. Let’s do it. For me*,* that was a fairly easy decision to leave. I leave easily because I want to see. I’m still trying to peek over that closed space of the war experience.*

Linda’s narrative illustrated how this yearning translated into advocacy when she encountered institutional barriers to travel and study:

… *there weren’t those scholarships at all. So*,* I was asking how we – from Serbia*,* from Bosnia*,* from former Yugoslavia – how we could get involved in those projects. I told him that there was a sanction*,* an embargo*,* that it was not there for us at all.*

#### Subordinate theme 3.3: preparedness for emergencies

Having once navigated life-threatening conditions, participants reported internalised survival strategies that remained accessible even decades later, whether a threat was real or hypothetical. A lasting sense of preparedness appeared to offer participants a degree of psychological control and a sense of competence in responding to potential crises.

... *what about the food*,* what about cigarettes*,* what about the water*,* what about the entertainment – I could not have believed that this could be an issue as well during those days. But also*,* entertainment*,* believe me*,* it’s crazy… I think it would be easier*,* but I really don’t want it. (Radek)*

#### Subordinate theme 3.4: condemnation of war

A pervasive motif was a critical and morally reflective stance toward war; war was consistently framed as a source of human suffering, hatred, and injustice. This condemnation extended beyond personal trauma, encompassing broader existential and ethical concerns, particularly regarding the sanctity of human life.

For Simone and Radek, the war experience prompted a loss of faith in humanity’s moral capacity. Despite this, both participants described posttraumatic growth through strengthened interpersonal relationships, suggesting that while trust in societal structures was eroded, faith in individuals remained intact.

... *basically losing some illusions that there is some sort of values of Western society that it will defend. Yeah*,* like the sanctity of human life*,* for example. And just like*,* people can be very*,* so to speak*,* in those wars that the worst in those people comes out. (Simone)*

Gabrielle offered a particularly reflective perspective on the role of hatred in wartime. She described how dichotomous narratives of good versus evil – ‘us’ versus ‘them’ – were imposed externally and also internalised as a means of psychological survival:

… *this hate towards the other*,* you know*,* we are good*,* they are bad*,* that’s the bottom line of the war and of the narrative and part of how you also survive*,* unfortunately.*

#### Subordinate theme 3.5: haunting of the past

Two enduring legacies of the war were persistent posttraumatic symptoms and experiences of prejudice. Emotional and physiological reactions to triggers such as airplanes, sirens, and media depictions of conflict remained powerful reminders of trauma:

*I was waking up for… quite a couple of months with these nightmares. Or I walked the street and I heard a normal civilian plane. It was already normal times. And this feeling: Oh my God*,* it’s coming again. (Radek)*

Participants also described experiences of prejudice and exclusion which traced back to war experience. For Simone such prejudice was particularly evident upon her arrival in Czech society which she experienced as unprepared for refugees:

*There was a feeling of basically just some kind of exclusion or… some kind of isolation. Like… this feeling that I understand that they don’t want me here*,* but I can’t do anything about it because I don’t have a choice anymore.*

Prejudice was perceived as an ongoing social dynamic. Gabrielle interpreted it as a lingering consequence of wartime isolation, manifesting today in subtle but systemic inequalities in opportunity. Radek emphasised the enduring presence of ethnic and intergroup prejudice within the Balkans, deeply rooted in the unresolved injustices and collective grievances that emerged during the war:

*I heard this*,* yeah: I cannot like you*,* I’m programmed to hate you and I’m sorry about it but it’s true.*

### Superordinate theme 4: relating to one’s roots

The experience of war and emigration reshaped participants’ sense of belonging and identity, prompting reflections on home, roots, and the meaning of origin. Their accounts revealed tensions between detachment and connection: while some felt estranged from national or ethnic labels, others described lasting cultural ties. Belonging emerged as both elusive and situational, influenced by migration, distance from family, and enduring associations with the Balkans.

#### Subordinate theme 4.1: ambivalent identity and the elusiveness of belonging

Emigration and war complicated the participants’ sense of identity, making the question ‘Where are you from?’ difficult to answer. Their narratives revealed emotional ambivalence, fragmented affiliations, and detachment from traditional national or ethnic labels:

*This feeling that I don’t belong anywhere. I am not Bosnian. I’m also not Serbian. I’m not Yugoslav. I don’t understand what that means. I think I was too small or I don’t really have a relationship that I can hold onto with that country. Am I Czech now? Am I an immigrant? Is that my identity? So… home is a very elusive category. (Gabrielle)*

#### Subordinate theme 4.2: emigration as opportunity and ambiguity

All participants viewed emigration as an opportunity – an escape from war and post-war stagnation and a chance to build a better life. However, their motivations varied. Gabrielle left to pursue a personal relationship and move beyond the emotional confines of war; Simone sought distance from hatred and violence; Radek saw emigration as a form of self-liberation; Linda made a pragmatic decision to improve her future prospects:

*Down there I had no chance*,* no opportunity*,* because the political situation has been changing very slowly. There’s no work. You can be as skilled as you want*,* but if you don’t have a chance to do something at that moment… and here*,* when the first opportunity came*,* we all just kind of… took the opportunity.*

Yet migration brought new challenges. Distance from family, particularly ageing parents, was a persistent emotional burden. Simone described migration as a process without clear endpoint:

… *just the migration*,* you know*,* it’s a never-ending process. Then it just comes back*,* the question of what to do with those people*,* or specifically my parents*,* you know*,* how they were left alone afterwards*,* so it was very complicated for me to organise it so I could be there.*

#### Subordinate theme 4.3: the Balkans as an emotional and cultural anchor

Despite ambivalence, Simone and Linda reflected a lasting connection to the Balkans as a region and space of warmth, emotional expressiveness, and cultural familiarity. This connection was not based on idealisation but on a recognition of shared temperament and lifestyle.

... *some things there are definitely more interesting and better*,* like the food*,* the weather*,* the interactions between people are much more intense than here. Here it’s a bit like*,* like a permanent spa (chuckles)*,* as I would say*,* not quite for us*,* like the interaction is not stimulating enough*,* I would say. (Simone)*

### Superordinate theme 5: transgenerational transmission

The final superordinate theme concerns the participants’ conscious efforts to transmit war-related experiences and survival knowledge to their children. This transmission was not accidental or implicit; rather, it was described as a deliberate process aimed at fostering resilience, awareness, and preparedness in the next generation.

#### Subordinate theme 5.1: preparing for socio-political awareness

Parents aimed to cultivate vigilance in their children, teaching them to recognise early signs of social or political instability. This awareness was seen as crucial for preventing future vulnerability and for increasing preparedness should similar conflict-related events arise in the future.

... *what are the signals in that society*,* whether it’s our own here or European or global*,* like what to look out for. Yeah*,* so that it doesn’t happen that one pays the price for not believing that it can happen. (Simone)*

#### Subordinate theme 5.2: teaching practical survival skills

In addition to preparedness, participants-parents emphasised passing on concrete survival competencies. Linda described a conscious intention to equip her children with basic competencies essential in future emergencies, thus ensuring continuity of self-sufficiency and resilience across generations:

… *I think about it*,* if*,* God forbid*,* something happens again*,* that I just*,* I have to*,* I* *must* *teach them some basic things*,* from how to cook*,* how to make something*,* how to sew…*.

## Discussion

In this study, we explored the understudied psychological dimensions of war and migration among civilians who survived the Balkan wars of the 1990s and subsequently rebuilt their lives abroad. Through in-depth, semi-structured interviews, we examined the narratives of four adults who were adolescents or young adults during the conflicts and whose migration was shaped by both political and economic consequences of the war. Employing IPA [20], we identified five superordinate experiential themes that illustrate how long-term psychological impact of war and war-related migration continue to reverberate across the life course in civilian populations.

Rather than discrete events, our findings suggest that war and migration are mutually entangled, evolving experiences that continue to shape identity, belonging, and intergenerational content decades later. This entanglement is reflected in the themes, which illuminate the complex processes through which participants make sense of their past, including their memories of war, sources of resilience during crisis, identity and value development across shifting cultural contexts, experiences of uprootedness, and intergenerational transmission of war-related knowledge and attitudes. In addition, they reflect participants’ moral reflections on human suffering, their lingering psychological responses to war-related adversity, and their encounters with prejudice and social exclusion in both wartime and migratory contexts.

Although several qualitative studies have examined war-related experiences, they have tended to focus primarily on military populations. For example, recent study explored the psychological well-being and reintegration challenges faced by veterans transitioning to civilian life after deployment [22]; other investigated the psychosocial consequences of conflict among internally displaced persons and military veterans in Ukraine [23]. Our study addresses the gap in research focusing specifically on the long-term, lived experiences of civilian survivors of war, specifically those who emigrated and navigated the aftermath of conflict-related hardships across sociocultural distances.

Our findings contribute to psychotraumatology research by suggesting that, for some survivors, war-related adversity and migration may function as an evolving psychological and social process that is continuously reinterpreted across the life span. Consistent with prior research on refugee and post-conflict populations [4, 24], our participants’ accounts highlighted how unresolved war memories and loss are not simply remnants of the past but are actively woven into present-day meaning-making and identity work. This is especially evident in the narratives marked by moral questioning, ambivalent attitudes toward belonging, and a heightened sensitivity to injustice – both past and ongoing. These accounts resonate with the concept of moral injury, which originated in research on military personnel but has since been applied to civilians who experience betrayal, loss of moral trust, or violations of deeply held ethical frameworks [25].

Importantly, participants’ narratives reflected both suffering and growth. Experiences of war and migration prompted critical reflection, shifts in personal values, and a heightened awareness of human interconnectedness. This aligns with research on posttraumatic growth in war-affected populations [26, 27]. Notably, the evidence of posttraumatic growth was found in post-war Sarajevo, particularly among younger individuals [26]. While contextual differences must be acknowledged, this underscores the dynamic nature of posttraumatic growth in those who, like our participants, experienced war in adolescence or early adulthood.

Moreover, the participants’ reflections demonstrate that psychological survival extends beyond individual coping to encompass collective, relational, and even transgenerational dimensions. A pronounced need for connection and not being alone was evident across all respondents. This finding aligns with the Tend and Befriend theory, which posits that affiliative behaviours emerge as adaptive responses to stress, fostering social bonding and support as a means of coping with threat [28].

Elements of an intergenerational dimension also emerged in participants’ accounts. Research on transgenerational trauma further suggests that the ways in which parents discuss and transmit life experiences can significantly shape their children’s coping capacities and psychological well-being into adulthood [29, 30]. Participants with children in our study primarily conceptualised transgenerational influence as the transmission of survival strategies and the deliberate cultivation of awareness; this is consistent with sociocultural models of intergenerational coping that emphasise the caregivers’ active role in passing on coping and meaning systems [31]. Our findings add nuance by showing that such transmission is not only about vulnerability but also about imparting ethical frameworks and relational strategies for navigating uncertainty.

Our findings also underscore how socio-political contexts shaped individual experiences of exclusion and continuity. Some participants recalled feelings of being marked as ‘the other’ both during the war and after migration – whether through ethnicised violence or subtle forms of discrimination in their host societies. These embodied experiences of marginalisation often compounded earlier conflict-related hardships and complicated the task of integration, further supporting models that emphasise the cumulative nature of stressors in exile [32]. These accounts further suggest that war-related distress is not static but may resurface or intensify at different points across the life span. The long-term sensitivity of war-related trauma has also been noted in recent literature: for instance, the isolation that resulted from the Covid-19 pandemic had a retraumatising effect on some survivors of the Yugoslav wars by reviving earlier feelings of wartime isolation [33].

Recognising this enduring vulnerability may have important implications for clinical practice. Trauma-informed interventions for civilian war survivors should integrate narrative, cultural and existential dimensions, recognising that recovery from traumatic stress is a lifelong and contextually embedded process. Evidence from other post-war populations suggests that meaning-oriented approaches and trauma-focused techniques, such as EFT and Matrix Reimprinting, may offer benefits in addressing long-term PTSD symptoms [34].

Beyond clinical interventions, these findings may offer insights relevant to migration and integration policies. Trauma recovery is not solely a clinical matter but also appears to be influenced by social and political processes, including the inclusiveness of host environments, recognition of migrants’ vulnerabilities, and community-level support. Participants’ accounts of experiencing persistent prejudice suggest that integration policies should extend beyond service provision to include anti-discrimination measures and support for hybrid identities. Culturally sensitive mental health services should be embedded in broader policies of integration, acknowledging that the aftermath of war and emigration continues to shape migrants’ identities, relationships, and sense of home.

### Limitations and future studies

While this study provides in-depth insights into the lived psychological consequences of war and migration, several limitations must be acknowledged. As with all IPA, findings were shaped by the researcher’s interpretative lens and complete neutrality is neither possible nor desirable. Although reflexivity was maintained throughout, future research might benefit from dialogical or collaborative approaches involving participants more directly in meaning construction.

Also, the sample size was small and purposively selected which limits generalisability. In addition, the sample was relatively homogeneous with regard to educational background, developmental stage, and resettlement context, as all participants were adolescents or young adults at the time of the war and ultimately migrated to the same host country. This deliberate homogeneity enhanced idiographic depth and information power [21] but may constrain the transferability of the findings to war-affected individuals who experienced the conflict at different life stages or followed different socioeconomic or migration trajectories. In line with IPA methodology, the study therefore aims for theoretical transferability rather than statistical generalisation [20].

While the present study achieved substantial analytic depth, future studies with larger or more diverse samples could extend these findings by exploring additional nuances across different conflict contexts. Comparative research including survivors of other wars or post-conflict settings would further enhance theoretical transferability. Nonetheless, the coherence, explicitness, and depth of the interviews in the present study enabled the identification of meaningful themes that illuminate the lived experiences of civilian survivors of the Balkan wars who emigrated abroad.

Moreover, since participants’ migration pathways were diverse – ranging from forced displacement to more voluntary emigration – future studies should examine how different migration trajectories shape long-term psychological adjustment, belonging, and resilience. Comparative designs contrasting those who remained in the region with those who migrated could further illuminate the interplay of trauma, integration, and transnational identity. Additionally, the Czech resettlement context may limit cultural transferability.

Finally, future quantitative or mixed-methods research could test the prevalence and predictors of the themes identified here – such as deliberate transgenerational transmission of survival knowledge, enduring crisis-preparedness, and ambivalent belonging – in larger, more socioeconomically diverse cohorts of Balkan and other war-affected civilians. Longitudinal designs and standardised measures of moral injury and posttraumatic growth would help determine whether these experiential patterns represent distinct long-term trajectories and how they are moderated by migration trajectory, host-society reception, and intergenerational processes. Second-generation perspectives should also be explored to better understand how war-related memories, coping strategies, and resilience are transmitted and reconstructed across generations. This would also contribute to migration studies more broadly, by showing how intergenerational transmission interacts with processes of diaspora formation, cultural adaptation, and integration in host societies.

## Conclusions

Our findings illuminate how war-related adversity and migration were experienced as dynamic, relational processes unfolding across the lifespan, contributing to a deeper understanding of trauma trajectories, identity reconstruction, and intergenerational adaptation. By centering the perspectives of civilians decades after conflict, this research highlights the value of holistic, culturally sensitive support systems. These insights may inform efforts to develop equitable policies and interventions to foster resilience in migrant and post-war communities, though further research is needed to explore their applicability in diverse contexts.

## Supplementary Information

Supplementary Material 1.

Supplementary Material 2.

## Data Availability

The data supporting the findings of this study are available upon reasonable request to the corresponding author.

## Abbreviations

IPA: Interpretative Phenomenological Analysis
PTSD: Post-traumatic Stress Disorder
