# Supplementary material for: Long-term psychological effects of war trauma and migration: an interpretative phenomenological analysis of Balkan war survivors
Source: BMC Psychol. 2026 Jan 29;14:253. doi: 10.1186/s40359-026-04033-3 (PMC12922406; doi:10.1186/s40359-026-04033-3)
Supplement: Supplementary file 1 — Supplementary Material 1. [file 40359_2026_4033_MOESM1_ESM.docx]

# Semi-structured interview guide

This interview guide was developed specifically for this study to explore participants’ wartime and migration experiences, coping mechanisms, and the long-term psychological effects. The questions are semi-structured to allow flexibility while ensuring comprehensive data collection. The phrasing or order may adapt based on the participant’s responses, but the core themes should be addressed.

## Wartime experience

- Can you tell me how you experienced the war? Are there particular moments or events that stand out to you?

## Coping mechanism and needs during the war

- What strategies or resources helped you cope with the challenges of the war?
- What did you miss that you think would have helped you get through this situation?

## Post-war coping and psychological impact

- In what ways do you feel the war has affected your emotional or psychological well-being?
- Have you experienced any ongoing signs of stress or trauma following the war? If so, could you describe them?
- Have you sought professional support (e.g., counselling or therapy) to address these effects? If so, could you share details about your experience?
- Have you encountered challenges with substance use (e.g., alcohol, drugs) following the war? If comfortable, could you elaborate?

## Long-term influence of wartime experiences

- Looking back now, what meaning do these experiences hold for you?
- In what ways do you think the war has changed you as a person?
- How have your wartime experiences influenced your values or worldview?
- How do these experiences shape the way you perceive or approach other life events?
- Have your wartime experiences influenced your relationships with family, partners, or children? If so, in what ways?

## Migration, identity and connection to home country

- When did you leave your home country? What was the impulse?
- How have your wartime experiences shaped your perception of your own identity?
- What is your relationship with the country you come from? How do you experience or feel when returning there?

## Current worldview

- Do you ever think about the possibility of a similar situation happening again? If so, do you have any thoughts or plans about how you would respond?

## Optional addition

- Is there anything we haven’t talked about that you feel is important to mention?
