# Supplementary material for: Long-term psychological effects of war trauma and migration: an interpretative phenomenological analysis of Balkan war survivors
Source: BMC Psychol. 2026 Jan 29;14:253. doi: 10.1186/s40359-026-04033-3 (PMC12922406; doi:10.1186/s40359-026-04033-3)
Supplement: Supplementary file 2 — Supplementary Material 2. [file 40359_2026_4033_MOESM2_ESM.docx]

| **Participant** | **Original transcript excerpt** | **Exploratory comments** | **Emergent theme** | **Subordinate theme** | **Superordinate theme** |
| --- | --- | --- | --- | --- | --- |
| **Radek** | *“I was waking up for quite a couple of months with these nightmares. Or I walked the street and I heard a normal civilian plane. It was already normal times. And this feeling: Oh my God, it’s coming again.”* | Sudden, intrusive re-experiencing decades later; ordinary sounds (plane) become triggers; time collapse – past danger feels immediately present; physiological arousal in safe context | Past intrudes into present through sensory triggers | Haunting of the past | Enduring imprints of war |
| **Gabrielle** | *“…we will never know what I would do or who I would be if I didn’t have that experience, but now that I do have it, it did colour my life in a very specific colour (…) that experience changed me completely… the way I experience the world… like a permanent threat that it can happen again.”* | War experience inseparable from current identity; fundamental shift in worldview; chronic anticipation of catastrophe, hypervigilance; war as an irreversible “colouring” of existence | War as enduring lens shaping identity and perception of threat | Preparedness for emergencies | Enduring imprints of war |
